# Recurrent Renal Colic in a Patient with Munchausen Syndrome

**DOI:** 10.3390/ijerph15040627

**Published:** 2018-03-29

**Authors:** Francesco Miconi, Valentina Rapaccini, Emanuela Savarese, Gabriele Cabiati, Augusto Pasini, Giovanni Miconi, Nicola Principi, Susanna Esposito

**Affiliations:** 1 Pediatric Clinic, Azienda Ospedaliera di Terni, 05100 Terni, Italy; francesco.miconi90@gmail.com (F.M.); manu-s84@hotmail.it (E.S.); gabrielecabiati@libero.it (G.C.); ocoman@tiscalinet.it (G.M.); 2 Pediatric Clinic, Università Tor Vergata, 00173 Rome, Italy; rapaccinivalentina@gmail.com; 3 Unità Sanitaria Locale (USL) Umbria 2, 05100 Terni, Italy; augusto.pasini@uslumbria2.it; 4 Università degli Studi di Milano, 20122 Milan, Italy; nicola.principi@unimi.it; 5 Pediatric Clinic, Department of Surgical and Biomedical Sciences, Università degli Studi di Perugia, 06100 Perugia, Italy

**Keywords:** factitious disorders, Munchausen syndrome, psychiatric diseases, renal colic, renal stones

## Abstract

Background: In most of the cases regarding children, factitious disorders (FDs) are intentionally produced by parents. Less attention is paid to FDs in which a child or adolescent intentionally induces or falsifies the disease to attain a patient’s role. Case presentation: A 13-year-old immigrated and adopted boy previously underwent an operation for renal joint syndrome and was affected by recurrent episodes of renal colic. The boy was admitted reporting acute left flank pain with scars on the mucous face of his prepuce and had a recent previous hospitalization for the same reason. Laboratory tests and radiological findings did not reveal any morphological or functional alterations. Self-induced FD was suspected, and a psychiatric consultation was performed. After psychiatric consultation and remission of the symptoms with a placebo, a diagnosis of Munchausen syndrome was suspected. The patient’s uncle was not initially convinced of the diagnosis. Some videos clearly showed that the boy was handling his prepuce to excrete stones, explaining the scars. A therapeutic plan with psychiatrist support was later accepted with a positive outcome. No further signs and symptoms of renal colic were reported. Conclusions: It is recommended that paediatricians include FD in the differential diagnosis of a persistent and unexplained medical condition. If suspicion arises, confirmation and long-term therapy by a group of qualified specialists, including psychiatrists, should be planned.

## 1. Introduction

Few conditions are as difficult to diagnose and treat as induced or falsified illnesses. In most of the cases regarding children, factitious disorders (FDs) are intentionally produced by parents, mainly the mother, to fulfil their own psychological needs, resulting in a form of child abuse [1]. Frequently, the perpetrator is psychiatrically ill with depression, personality disorder, or prior personal history of somatoform or factitious disorder. Moreover, the relationship between the mother and the child is frequently characterized by disproportion and a bond of caring [2].

Clinical presentation can vary significantly. However, each of these cases is generally identified as a case of FD by proxy, which is alternatively named Munchausen syndrome by proxy. The name is derived from the history of Baron von Munchausen, an 18th century German officer who was known for embellishing his personal history with a great number of incredible and dramatic events [3]. Similarly, all the children with Munchausen syndrome by proxy undergo numerous medical visits and laboratory and radiological tests, are frequently hospitalized and receive costly and potentially harmful therapies. The risk of morbidity and mortality of these patients is generally significantly increased. 

Given that it is a very dangerous condition, Munchausen syndrome by proxy is frequently reported and discussed in the paediatric literature where it is commonly highlighted that illness falsification by caregivers must be considered when children present with persistent, unexplained symptoms or laboratory findings [1]. This protocol favours the identification of abused children and the implementation of all measures to confirm the diagnosis and initiate therapy and legal procedures when needed. Significantly less attention is paid to the FDs in which it is the child or adolescent himself that intentionally induces or falsifies the disease to attain a patient’s role without any obvious gain. [4]. Generally, this condition occurs in older children and adolescents who already suffer from medical conditions requiring treatment and hospitalization and have a history of environmental or family deprivation and emotional and physical abuses [5,6].

When self-induced FDs are not promptly recognized, the final prognosis can be poor. Together with the health problems associated with hurting themselves by causing symptoms and the risk related with multiple tests, procedures and treatments, subjects with self-imposed FD are at a high risk of significant exacerbation of the existing psychiatric problem. Suicide has been described [7,8,9]. Prompt identification of these cases should be necessary. Unfortunately, prompt identification rarely occurs. Because the index of suspicion is low, clinicians confuse FDs with somatization. In addition, even when considered directly responsible for the clinical problems for which they seek treatment, patients firmly deny any responsibility. This paper describes a case of an adolescent boy with a history of mild ureteropelvic junction obstruction leading to several hospitalizations and surgical repairs. The boy later attempted to insert stones into his urethra and simulate urolithiasis to justify unlikely renal colic.

### Case Presentation

A 13-year old boy born in Albany was admitted to Santa Maria Hospital of Terni on 19 July 2017 for left renal colic. He had been an orphan at the age of three years and was entrusted and adopted to his uncles in Italy at the age of four years. His clinical history indicates no relevant medical problems until the age of nine when he underwent appendectomy for acute appendicitis. In the following two years, no disease occurred, and neuromotor and psychiatric development was normal as stated by the primary care paediatrician who visited him during annual health check-ups.

Approximately one year before admission to our hospital at 12 years of age, the boy began to suffer from severe pains in the left lumbar region. These painful crises were characterized by monthly cadence, sudden onset, extremely high intensity and spontaneous remission within a few tens of minutes. To assess whether the pain depended on the kidney and the urinary tract, renal ultrasonography and renal scintigraphy with technetium-m99 mercaptoacetyltriglycerine (MAG 3) were performed. Echography revealed a grade 3 hydronephrosis (20 mm) in the left kidney. Almost all calyces were observed, and a large renal pelvis without parenchymal thinning was noted. MAG3 revealed good renal function balanced between the two kidneys with only slight reduction of the ureteropelvic drainage on the left side. A ureteropelvic junction obstruction caused by abnormal polar vessels was suspected. A JJ stent was positioned. A few weeks later, laparoscopic robotic pyeloplasty was performed. However, no polar vessels were found.

Despite this procedure, two months later, he was admitted to a second hospital for left renal colic. Here, radiographic evaluation of the abdomen revealed that the stent was correctly positioned, and renal ultrasonography revealed reduction of the known left pelvic dilation to 17 mm. The abdominal pain was attributed to the prolonged stay of the JJ stent with a supposed coexistence of vesicoureteral reflux caused by the stent itself. No complication of the pyeloplasty was noted. The JJ stent was removed, and this procedure was associated with the apparent remission of pain. 

However, new hospitalization in a third hospital was necessary on July 2017 for a new episode of renal colic. During this hospitalization, a further reduction of the pelvic dilation to 10 mm was noted through renal ultrasonography and abdominal magnetic resonance. Paradoxically, repeated urinalyses never evidenced blood or increased mineral excretion suggesting urolithiasis. Tests for the evaluation of renal function were in a normal range. The boy was discharged without any therapy but with the recommendation to return within two weeks for further assessments. 

**Figure 1:**
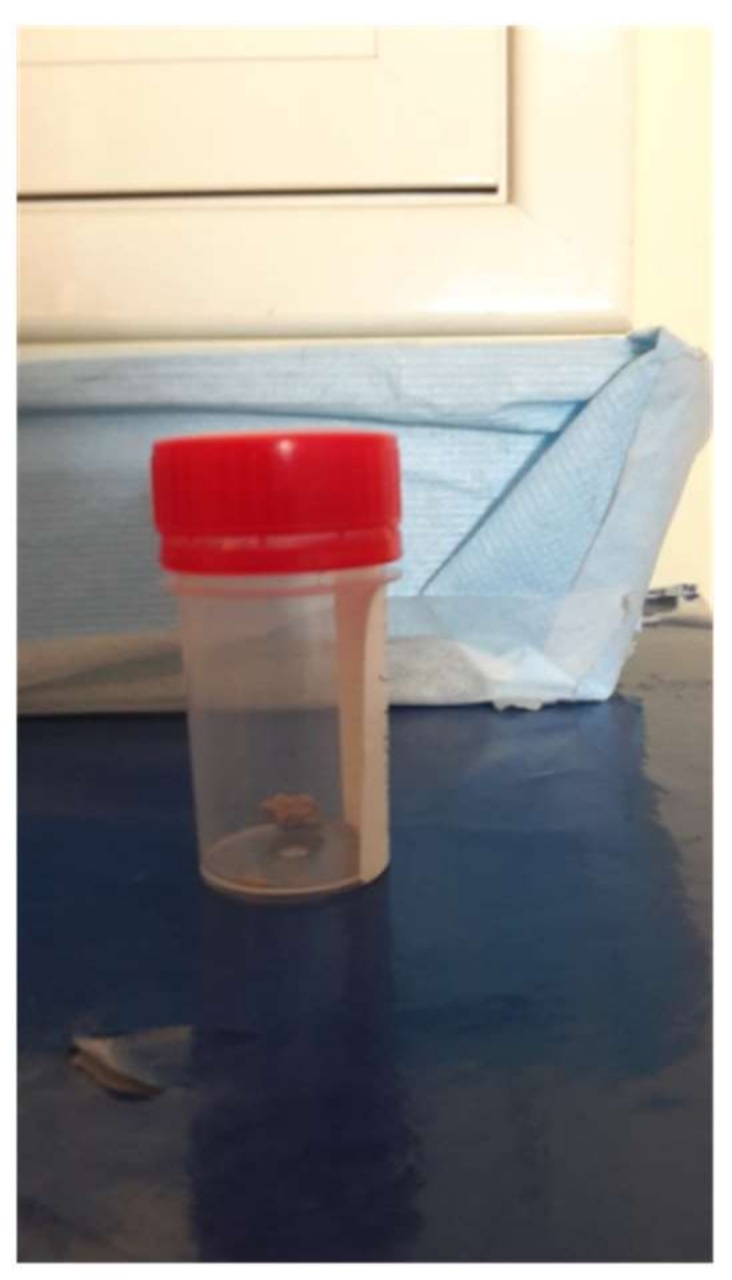
A sample of what the patient referred to as his urinary stones.

One day after discharge, symptoms of renal colic occurred, and he was admitted to our hospital. Physical examination did not reveal significant abnormalities. Only some small scars were noted on the mucous side of the prepuce. When asked, the boy could not provide an answer regarding the origin of these scars and how long they were present. All the laboratory tests already performed, including urinalysis, were repeated. In the repeated analysis, no abnormal results were observed. In particular, no blood was detected, whereas Mg, Ca, P, oxalate, citrate, sulfate and ammonia excretion levels were in the normal range. Radiopaque concretions were not noted in direct abdomen radiography. Ultrasonography was negative for calculi, and the previously observed slight pielectasia in the left renal pelvis was confirmed. The bladder was distended without appreciable endoluminal images. Despite these findings, on the morning of the second day of hospitalization, the boy indicated he expelled multiple stones from the urinary tract during the night. The stones were greyish-whitish with irregular margins and seven to eight millimetres in diameter (Figure 1). Laboratory evaluation revealed that the examined stone material was not related to lithiasis of the urinary tract. 

Thus, the patient was observed during his following urinations by the nursing staff to monitor the possible expulsion of additional stones. After three days of close observation and surveillance of each urination event, the patient did not expel any additional stones.

Despite this, he continued to claim he experienced severe pains in his left flank. Suspicions about the factitious nature of his symptoms were raised. A placebo was administered, which led to the resolution of renal colic. Consequently, a psychological and psychiatric evaluation of the boy was performed. Regarding the neuropsychiatric evaluation, several specific tests and interviews were performed. The Wechsler Intelligence Scale for Children: Fourth Edition (WISC: IV) was used to assess intelligence and cognitive function. From the evaluation, a cognitive level in the standard (QI of 80) emerged with a Verbal Comprehension Index (VCI) of 108, a Perceptual Reasoning Index (PRI) of 93, a Working Memory Index (WMI) of 103, and a Processing Speed Index (PSI) of 53. To examine the presence of anxious symptoms, Multidimensional Anxiety Scale for Children (MASC) was used. Clinically significant scores were noted in areas related to Performance Fears (T80) and Physical Symptoms (T72). To analyse the presence of depressive symptoms, Children’s Depression Inventory (CDI) was used, and clinically positive scores were found in areas related to Negative Self Esteem (T75), Interpersonal Problems (T80) and Anhedonia (T71). Overall, based on tests and interviews, socio-relational difficulties and a marked tendency for isolation emerged that was associated with relevant aspects of emotional and behavioural inhibition. Therefore, individual psychotherapy intervention was recommended. The paediatric psychiatrist confirmed the diagnosis of Munchausen syndrome according to the DSM 5 diagnostic criteria for this condition [10].

The patient’s uncle was not convinced of the diagnosis and pretended to demonstrate the real nature of the patient’s symptoms by showing some videos in which the patient expelled the stones from his urethra during urination. However, upon closer observation of the videos, it appeared clear that the boy was actually handling his prepuce to excrete the stones. This evidence explained the scars that were found on the mucous face of his prepuce. Despite this information, the patient’s uncles continued to refuse the diagnosis. Fortunately, a therapeutic plan with psychiatrist support was later accepted with a positive outcome. No further signs and symptoms of renal colic were reported. 

## 2. Discussion

The prevalence of self-induced FDs in paediatric populations is unknown. Most of the available data have been collected in psychiatry consultation-liaison services and probably underestimate the true incidence of this condition in the general paediatric population. In addition, ethnic and cultural factors may influence self-induced FDs prevalence. It is highly likely that some of the less severe cases not followed in psychiatric centres are lost. Moreover, in some cases, the diagnosis is not made because the clinician can consider reported signs and symptoms as somatization instead of falsification. A systematic survey evaluating self-induced FD in children referred to a large multidisciplinary tertiary-care child health centre in Germany evidenced that in 2003–2005, the prevalence was only 0.03% among patients aged 6–18 years [11]. Similar to adults, FDs are more common in girls compared with boys and occur mainly in older children and adolescents [12]. Clinical manifestations can vary. 

In a single subject, psychological or physical signs and symptoms can prevail or coexist with the same evidence. Although some subjects can exhibit multiple signs and symptoms, generally only one body system is involved in the physical lesions that can be simulated or directly produced. The skin is the most common site of lesions. Urinary tract involvement is rare. Some cases of urine tampering to simulate hematuria [13] or proteinuria [14] have been described. However, in 1976, Sneed and Bell published a case quite similar to that reported here [15]. In both cases, attempts to insert stones into the urethra to simulate urolithiasis were made. 

Although less common than other psychiatric diseases, such as somatoform and dissociative disorders, self-induced FDs in children and adolescents are a significant psychiatric problem. Frequently, particularly in cases affecting adolescents with predominant psychiatric manifestations, FDs are part of a more complex clinical picture, including abnormal personality, perceptual abnormalities (i.e., hallucinations and disturbances of thought process or thought content), and suicide and homicidal tendencies [16]. The causes of self-induced FDs are difficult to determine. In some patients in whom magnetic resonance imaging demonstrated brain abnormalities, it was hypothesized that a congenital or acquired structural alteration of the central nervous system could be associated with the development of a self-induced FD [17]. In agreement with this supposition is the finding that reduced frontal lobe and left orbitofrontal cortex volume can be detected in a significant number of individuals who develop borderline personality disorder or complex post-traumatic stress disorder following early emotional trauma. On the other hand, the history of patients with FD is frequently full of dramatic events. This finding is common among adults who have suffered from traumatic events and were repeatedly hospitalized for real clinical problems in most of self-induced FD cases [18]. Moreover, in some adult patients, a previous history of living in a foster home, disturbing family disharmony, physical and sexual abuse, and early losses can be detected [19]. In children, a dramatic early biography in many self-induced FD cases is frequently evidenced. In some patients, a history of Munchausen syndrome by proxy may even be present. Ehrlich et al. found that approximately 42% and 50% of subjects with self-induced FD suffered an abuse or lived in foster care/were adopted, respectively [11]. The case of self-induced FD described here exhibits most of the characteristics mentioned above. He was born in Albany, was an orphan, was adopted by an uncle at four years of age and moved to Italy; thus, his daily habits and reference persons were changed. Moreover, he had a renal problem for which he had to endure numerous medical visits, radiological and laboratory tests, repeated hospitalizations and surgical procedures. Based on these premises and the evidence that early emotional and physical stress is associated with a high risk of later psychiatric and psychological problems [20], it is not surprising that the boy described here developed self-induced FD and a care-eliciting behaviour. Interestingly, as previously reported in adults [19,21], the self-induced FD involved a body system of which he had great knowledge. Clinical manifestations of renal colic, including pain, were well known as the close relationship between pain and urolithiasis. Thus, he was able to simulate the clinical picture of renal colic. Longitudinal data on FDs are scare, particularly in children. However, available data seem to indicate that contrary to adults who are at increased risk of a negative evolution, children frequently admit to their deceptions and are at low risk for repetition, particularly when the falsehood is immediately discovered. In this regard, a fundamental role is played by caregivers. In some cases, such as the case reported here, caregivers can initially refuse diagnosis and oppose psychotherapy. However, to avoid the risk of negative evolution, early diagnosis is necessary. 

## 3. Conclusions

It is recommended that paediatricians include FD in the differential diagnosis of a persistent and unexplained medical condition along with somatization, malingering, and Munchausen’s by proxy abuse. Clinical manifestations that occur on an easily accessible part of the body or when the child is alone or unobserved, such as the elimination of stones in urine as noted in this case, should arouse suspicion. A detailed analysis of the patient history with particular attention to all the traumatic events that are typically associated with development of abnormal behaviour must be performed. We think that in these cases it is extremely important to discuss the diagnosis of FD with caregivers. Finally, if suspicion arises, we recommend to plan long-term therapy by a group of qualified specialists, including psychiatrists. Committed participation of caregivers not involved in previous abuse is essential.

## References

[1] Flaherty E.G., Macmillan H.L. (2013). Committee on Child Abuse and Neglect. Caregiver-fabricated illness in a child: A manifestation of child maltreatment. Pediatrics.

[2] Bass C., Jones D. (2011). Psychopathology of perpetrators of fabricated or induced illness in children: Case series. Br. J. Psychiatry.

[3] Asher R. (1951). Munchausen’s syndrome. Lancet.

[4] Jaghab K., Skodnek K.B., Padder T.A. (2006). Munchausen’s syndrome and other factitious disorders in children: Case series and literature review. Psychiatry.

[5] Yates G.P., Feldman M.D. (2016). Factitious disorder: A systematic review of 455 cases in the professional literature. Gen. Hosp. Psychiatry.

[6] Caselli I., Poloni N., Ielmini M., Diurni M., Callegari C. (2017). Epidemiology and evolution of the diagnostic classification of factitious disorders in DSM-5. Psychol. Res. Behav. Manag..

[7] Bliss E.L. (1974). Self-induced abscesses: A diagnostic and treatment enigma. Int. J. Psychol. Med..

[8] Sale I., Kalucy R. (1980). An observation on the genesis of Munchausen’s syndrome: A case report. Aust. N. Z. J. Psychiatry.

[9] Paperny D., Hicks R., Hammar S.L. (1980). Munchausen’s syndrome. Am. J. Dis. Child..

[10] American Psychiatric Association (2013). Diagnostic and Statistical Manual of Mental Disorders.

[11] Ehrlich S., Pfeiffer E., Salbach H., Lenz K., Lehmkuhl U. (2008). Factitious disorder in children and adolescents: A retrospective study. Psychosomatics.

[12] Libow J.A. (2002). Beyond collusion: Active illness falsification. Child Abuse Negl..

[13] Salmon R.F., Arant B.S., Baum M.G., Hogg R.J. (1988). Factitious hematuria with underlying renal abnormalities. Pediatrics.

[14] Tojo A., Nanba S., Kimura K. (1990). Factitious proteinuria in a young girl. Clin. Nephrol..

[15] Sneed R.C., Bell R.F. (1976). The Dauphin of Munchausen: Factitious passage of renal stones in a child. Pediatrics.

[16] Kocalevent R.D., Fliege H., Rose M., Walter M., Danzer G., Klapp B.F. (2005). Autodestructive syndromes. Psychother. Psychosom..

[17] Aouillé J., Rouillon F., Limosin F. (2003). Factitious anemia and magnetic resonance imaging abnormalities. Can. J. Psychiatry.

[18] Sadock B.J., Sadock V.A., Ruiz P. (2014). Kaplan and Sadock’s Synopsis of Psychiatry: Behavioral Sciences/Clinical Psychiatry. Factitious Disorders.

[19] Kapfhammer H.P., Rothenhäusler H.B., Dietrich E., Dobmeier P., Mayer C. (1998). Artifactual disorders—Between deception and self-mutilation. Experiences in consultation psychiatry at a university clinic. Nervenarzt.

[20] Pechtel P., Pizzagalli D.A. (2011). Effects of early life stress on cognitive and affective function: An integrated review of human literature. Psychopharmacology.

[21] Sutherland A.J., Rodin G.M. (1990). Factitious disorders in a general hospital setting: Clinical features and a review of the literature. Psychosomatics.

